# Flaxseed Cake as a Tool for the Improvement of Nutraceutical and Sensorial Features of Sourdough Bread

**DOI:** 10.3390/foods9020204

**Published:** 2020-02-16

**Authors:** Chiara Sanmartin, Isabella Taglieri, Francesca Venturi, Monica Macaluso, Angela Zinnai, Silvia Tavarini, Asia Botto, Andrea Serra, Giuseppe Conte, Guido Flamini, Luciana G. Angelini

**Affiliations:** 1 Department of Agriculture Food Environment, University of Pisa, via Del Borghetto 80, 56124 Pisa, Italy; chiara.sanmartin@unipi.it (C.S.); isabella.taglieri@for.unipi.it (I.T.); monica.macaluso@phd.unipi.it (M.M.); angela.zinnai@unipi.it (A.Z.); asia.botto@agr.unipi.it (A.B.); andrea.serra@unipi.it (A.S.); giuseppe.conte@unipi.it (G.C.); luciana.angelini@unipi.it (L.G.A.); 2 Interdepartmental Research Center “Nutraceuticals and Food for Health”, University of Pisa, Via del Borghetto 80, 56124 Pisa, Italy; guido.flamini@unipi.it; 3 Department of Pharmacy, University of Pisa, Via Bonanno Pisano 6, 56126 Pisa, Italy

**Keywords:** nutraceuticals, antioxidants, bioactive compounds, PUFAs, MUFAs, sourdough bread, flaxseed, fortification

## Abstract

Flaxseed has been recently studied for the formulation of healthy functional foods that are also useful for the prevention of chronic diseases. In this context, the production of sourdough bread fortified with different percentages of flaxseed cake was performed and the interactions among the bioactive compounds derived from both sourdough and flaxseed cake were investigated. The organoleptic properties as well as nutraceutical and chemical characteristics regarding pH, ethanol, lactic and acetic acid content, fatty acids profile, the concentration of total polyphenols, antioxidant capacity, and aroma volatile organic compounds were determined to evaluate the efficacy of leavening in the different matrices in comparison with the traditional bread. The results obtained demonstrated that flaxseed cake-enriched sourdough bread can represent a potential vehicle for bioactive compounds with the possibility of obtaining high-quality products with improved nutritional profiles and desired health attributes. Furthermore, the bread obtained with the addition of 7.5% of flaxseed cake was individuated as the best formulation to produce sourdough bread fortified with flaxseed cake by the overlap between three series of information coming from physical-chemical, nutritional, and sensorial analyses. In conclusion, in the operating conditions adopted, the use of flaxseed cake could represent a viable alternative for the production of fortified bread based on sourdough technology.

## 1. Introduction

The use of sourdough as a biological leavening agent is one of the oldest biotechnological processes in traditional cereal food production and still plays an important role in bread making [1,2,3,4]. Nowadays, the literature is very rich in reports that show how sourdough fermentation may affect the functional features of leavened baked goods. The use of sourdough as a leavening agent allows us to obtain particular characteristics of bread in terms of texture, palatability, and nutritional values, as well as upgrading its shelf life [5,6,7,8,9,10,11]. In recent years, the traditional sourdough bread production has gained tremendous success with rising demand by consumers for more organic, tasty, and healthy foods [6]. Consumers increasingly request functional foods, taking into account their higher content in nutraceutical compounds and their direct contribution in preventing nutrition-related diseases [12,13,14]. Therefore, supplementing bread with nutritious additives in order to boost its physical and nutritional properties [15,16], as well as the use of composite flour for improving bread protein quality, are increasing practices [16,17,18,19,20,21,22,23]. At present, there is a growing request for new sources of bioactive ingredients suitable for the development of innovative functional products [24,25]. In this context, according to a circular economy concept, food byproducts (i.e., peptides, carotenoids, and phenolic compounds) could be an interesting and cheaper source of potentially functional ingredients [25,26].

Recently, in the health food market, increasing attention has been paid to products and co-products deriving from flaxseed (*Linum usitatissimum* L.), as promising functional foods and ingredients. It is well known, in fact, that the seeds and oil of interesting oilseed crops represent a rich source of bioactive compounds that have positive effects on disease prevention [27,28,29,30]. The nutritional importance of flaxseed is justified by its content of proteins (22%), lipids (43%) and minerals (3%). In particular, flax protein is characterized by a relative richness in arginine, aspartic acid and glutamic acid; flax oil is also an important source of omega-3 fatty acids, especially α-linolenic acid (ALA) (more than 50% of the total fatty acids). Flaxseed also contains a huge amount of lignans, such as secoisolariciresinol diglucoside, known for their health benefits, and fiber, such as cellulose, mucilage gums, and lignin. After screw-pressed oil extraction, a huge amount of pressed flaxseed cake remains as a valuable byproduct. Even if it is mainly used as a cattle feed, flaxseed cake could find interesting application in the food, cosmetic and pharmaceutical industries, thanks to its interesting nutrient profile and functional properties, such as a good protein content (about 36% with 85% digestible), residual oil (from 7% to 10%) and other minor molecules, such as phenolic acids and flavonoids [31]. For the above-mentioned properties, flaxseed cake has immense usable potential as an ingredient for the food industry, particularly as an additive in baking products [32]. Flaxseed cake is gluten-free and, therefore, it can lower the total gluten content of the flour mix when used as an alternative raw material [31]. In addition, its considerable amount of proteins, omega-3, omega-6, and minerals is of special interest in gluten-free bread, since it is known that the gluten-free diet can be low in fiber and minerals (iron, zinc, magnesium, and calcium), and may contain excess saturated fats [32,33].

Several studies report the possibility of obtaining value-added food products by flaxseed (seed and cake) incorporation in baker’s yeast bread, pointing out the improvement of physical, sensory, and nutraceutical characteristics [31,33,34,35,36,37], but, to the best of our knowledge, no studies have been carried out on sourdough bread fortified with flaxseed cake.

Therefore, considering the potential health benefits of flaxseed and the high nutritional and nutraceutical value of sourdough bread, together with the increasing inclination of consumers towards healthy food, the aim of the present research is to investigate the technological properties of sourdough bread fortified with pressed flaxseed cake. The organoleptic properties, as well as chemical characteristics regarding pH, ethanol, lactic and acetic acid content, fatty acids profile, the concentration of total polyphenols, antioxidant capacity and aroma volatile organic compounds, were determined to evaluate the efficacy of leavening in the different matrices in comparison with the traditional bread.

An innovative approach based on the overlap between three series of information coming from physical–chemical, nutritional and sensorial analyses was applied to individuate the best formulation to produce sourdough bread fortified with flaxseed cake.

## 2. Materials and Methods

### 2.1. Reagents and Standards

Folin–Ciocalteu reagent was purchased from Merck (Darmstadt, Germany). Water was purified by a Milli-Q water purification system from Millipore (BurlingtonBedford, MA, USA). Other reagents, including methanol (HPLC grade), ABTS, and DPPH, were purchased from Sigma Aldrich (St. Louis, MO, USA).

### 2.2. Raw Material

The seeds used in this study were brown seeded, belonging to Sideral variety. They were produced organically in an on-farm trial, carried out in the 2017–2018 growing season in the lowland area (latitude 43°40′48′’N, longitude 10°30′1′’E) of the Pisa Province (northern Tuscany, Italy). Flaxseed cake was obtained after oil extraction by cold pressing, ground and stored at −20°C in a sealed vacuum container until the analysis and processing.

The sourdough utilized during the study was supplied by Dolcezze Savini Srl (Via S.Aleramo, 24/26-50063-Figline Valdarno (FI)) while the wheat flours used for the refreshment procedure (hard wheat flour type 0) and the bread making (weak wheat flour type 0) were provided by Molino F.lli Giambastiani Srl (Via Nazionale del Brennero, 798-55029-Ponte a Moriano (LU)).

Both flour type 0 and flaxseed cake were chemically characterized in terms of dry matter percentage, water activity, free acidity, and phytochemical properties. The results obtained from these assays are reported in Table 1.

Refreshment procedure of starter dough, as well as baking protocol and operating conditions (time and temperature) adopted in the storage of starter dough, in bulk fermentation and in cooking phases, were performed as described in a previous paper [3]. Bread making tests were conducted at the Food Technology laboratory of the Department of Agriculture Food and Environment of Pisa University; moreover, for each formulation, three replications were performed.

Different formulations of sourdough bread (water 32%; sourdough 16%, flour 52%) were produced using flaxseed cake flour at different percentages (Table 2).

### 2.3. Chemical–Physical Characterization

Chemical–physical analysis of flour, dough, and bread were performed following AACC standard methods for moisture [38], pH [39], free acidity [40], and volume [41], while water activity was measured by HygroPalm HP23-AW-A (Rotronic AG, Grindelstrasse 6 CH-8303 Bassersdorf, Switzerland). The concentration of the main fermentative metabolites (ethanol [42], L-lactic acid [43], D-lactic acid [44], acetic acid [45]) was determined by using specific enzymatic kits (Megazyme Ltd.), after pre-extraction with Carrez I and II solutions. The aromatic profile of control and fortified breads was analyzed by headspace solid phase microextraction gaschromatography-mass spectrometry HS–SPME–GC/MS [14].

#### Color Determination

Crumb color of baked samples as a function of the formulation was quantified using a benchtop tristimulus colorimeter (Eoptis, Mod. CLM-196 Benchtop, Trento, Italy) supplied with its own white reference standard. Crumb samples were taken from the two center slices of the loaf; in particular, the surface area analyzed was about 24 cm^2^ for each determination. Color was evaluated on the basis of the CIE L*a*b* color System accepted by the Commission International Eclairage, where L* is the lightness, a* and b* are the red–greenness and blue–yellowness components, respectively.

The results were expressed as metric distances among the chromatic coordinates ($∆E_{ab}^{*}$) values by the following equation:

(1) $$∆E_{ab}^{*}=\sqrt{∆L^{*}{}^{2}+∆a^{*}{}^{2}+∆b^{*}{}^{2}}$$

where: ∆L* = L_1_ − L_0_; ∆a* = a_1_ − a_0_; ∆b* = b_1_ − b_0_.

### 2.4. Total Phenols, Flavonoids and Anti-Radical Activity of Linseed Cake, Flour, and Breads

#### 2.4.1. Extract Preparation

Samples (0.5 g) were extracted with 10 mL of 80% methanol. The mixture was sonicated for 30 min and centrifuged (15 min, 3500 rpm). The supernatant was filtrated with a syringe filter (0.45 μm), recovered, and stored at 4 °C.

#### 2.4.2. Total Phenols Evaluation

Total phenols concentration was determined according to Tavarini et al. [46]. Results were expressed as milligrams of gallic acid equivalents (GAE) per gram of sample (dm).

#### 2.4.3. Total Flavonoid Evaluation

Total flavonoids were quantified by the aluminum chloride colorimetric method, following the procedure reported by Kim et al. [47]. Absorbance was read at 510 nm and results were expressed as mg of catechin equivalents (CAE) per gram of sample, using a standard curve of catechin.

#### 2.4.4. Determination of Anti-Radical Activity

Traditional assays provide only an estimation of the real antioxidant potential of the extracts, so the free anti-radical activity of flaxseed cake, flour 0, and bread sample was evaluated by means of two different methods: the DPPH free radical method, according to Tadhani et al. [48]; and a 2,2′-azino-bis(3-ethylbenzothiazoline-6-sulphonic acid antioxidant assay (ABTS), as reported in a previous paper [49]. The results were expressed as µmol Trolox equivalents (TE) per gram of sample, using a standard curve of Trolox, in the range of 0–200 µmol L^−1^ for the DPPH assay and 0.2–1.5 mM range for ABTS.

### 2.5. Volatile Organic Compounds Characterization

#### 2.5.1. Headspace Solid Phase Microextractions (HS–SPME)

The headspaces of the bread samples (whole and sliced) were collected by solid phase microextraction according to [13,14]. The adsorption of the volatile analytes was performed with a Supelco divinylbenzene/carboxen/polydimethylsiloxane (DVB/CAR/PDMS) assembly (50/30 μm coating thickness, St. Louis, MO, USA) preconditioned according to the manufacturer’s instructions.

All the SPME sampling and desorption conditions were identical for all bread samples, which were placed into glass containers closed with aluminum foil. After 30 min of equilibration time, the foil of each container was perforated by the holder (syringe), and the fiber exposed to the headspace of the sample for 30 min at room temperature.

#### 2.5.2. Gas Chromatography–Mass Spectrometry Analyses and Peak Identification

Gas chromatography–electron impact mass spectrometry (GC–EIMS) analyses were performed with an Agilent 7890 B gas chromatograph (Agilent Technologies Inc., Santa Clara, CA, USA) equipped with an Agilent HP-5MS (Agilent Technologies Inc., Santa Clara, CA, USA) capillary column (30 m × 0.25 mm; coating thickness 0.25 μm) coupled with an Agilent 5977 B single quadrupole mass detector (Agilent Technologies Inc., Santa Clara, CA, USA), according to [13,14]. The characterization of the volatiles was based on the comparison of their retention times (t_R_) with those of pure reference compounds and their linear retention indices (LRIs), determined relative to the t_R_ of a series of *n*-alkanes. Their mass spectra were compared with those listed in the commercial libraries NIST 14 and ADAMS, as well as in a homemade mass-spectral library, built up from pure substances and components of known samples and MS literature data [13,14].

### 2.6. Sensory Characterization (Crust and Crumb)

Sensory profiles of the bread samples were determined by descriptive analysis by a panel of trained assessors (10 assessors, 6 females and 4 males, aged between 23 and 60 years). All the involved assessors were included in the “expert panel” of the Department of Agriculture, Food and Environment (DAFE) of the University of Pisa and the DAFE internal procedure for assessor selection and training was applied, as reported in a previous paper [50].

Starting from this general protocol, a specific training section based on the “Procedure for sensory evaluation of bread”, developed for the trained panel by Elia [51], was further organized for all the selected panelists before the starting of the specific tasting sessions. The second part of the specific training was aimed at the design of the method specific for the sensory evaluation of bread fortified with flaxseed cake and all the trained panelists were also involved in a consensus panel specifically aimed at the generation of descriptors and their definitions.

Starting from the lists of attributes previously developed by Heenan and coworkers [52] and Elia [51], a final set of 32 descriptive parameters for sourdough bread evaluation, including both quantitative and hedonic attributes, was individuated by agreement among panelists (Table 3).

The panelists always had the option to include relevant observations specific for flaxseed flour under an “others” parameter; e.g., flax, chestnut, barley, nut, and hay were considered very specific items that could be included in this category as they were only detected in a few samples and therefore did not warrant being included in the final set, which was arrived at by consensus.

Tasting was carried out according to the protocol previously developed and validated [51]. All the sections were arranged in the morning, in a well-ventilated quiet room and in a relaxed atmosphere. All samples were assessed 2 h after to be taken out of the oven. A 20 g portion of each sample was randomly labeled with a three-digit numeric code and provided to assessors in a double-blind presentation to avoid any expectation error [53]. The samples were presented in a different order at each tasting session and 10 min intervals were allowed between each sample. Furthermore, a bread sample was randomly replicated to verify the performance of the panel at each tasting session. For evaluation, each assessor was provided with filtered water and asked to cleanse their palate between tastings.

**Figure 1:**
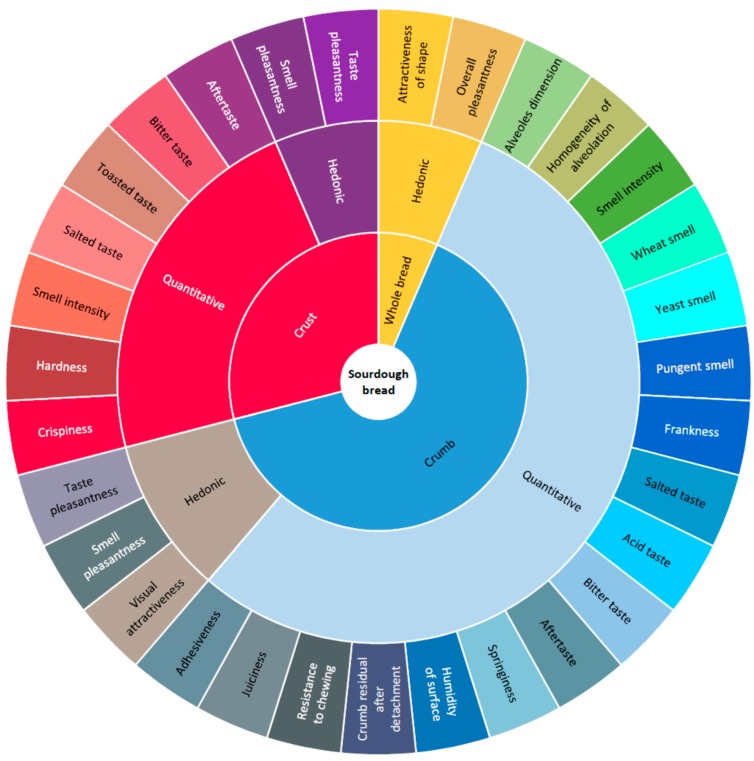
Sensory wheel of cooked breads (XLSTAT version 2019.4.1).

In order to evaluate the breads as a function of fortification, the panelists rated the intensity of each parameter (Figure 1) from 0 (minimum scale) to 9 (maximum scale), including visual, aroma, and taste attributes, of crust and crumb separately as well as some hedonic parameters in order to provide some indications about whole quality of the tasted breads.

### 2.7. Fatty Acid Profile Characterization

An acid trans-methylation was used to prepare fatty acids for the analysis following the procedure proposed by Christie [54] with some modifications. Briefly, fatty acid methyl esters (FAME) were prepared by pouring 5 g of sample and 4.5 mL of 10% HCl methanolic solution into a 20 mL vial and mixed with a vibration mixer for 60 s. A nonadecanoic acid (1 mg) was added to the mix as an internal standard. After 8 h, 5 mL of n-hexane were poured into the vial and the mixture was shaken for 1 min. The layers were allowed to separate, and the hexane fraction was injected into a GC2010 Shimadzu gas chromatograph (Shimadzu, Columbia, MD, USA) equipped with a flame-ionization detector and a high polar fused-silica capillary column (Chrompack CP-Sil88 Varian, 152 Middelburg, the Netherlands; 100 m, 0.25 mm i.d.; film thickness −1, 0.20 μm) for gas-chromatographic (GC) analysis. Hydrogen was used as the carrier gas at a flow of 1 mL min used with a split ratio of 1:40. An aliquot of the sample was injected under the following GC conditions: the oven temperature started at 40 ◦C and held at that level for 1 min; it was then increased to 163 °C at a rate of 2 °C/min, and held at that level for 10 min, before being once again increased to 180 °C at 1.5 °C/min and held for 7 min, and then to 187 °C at a rate of 2 °C/min; finally, the temperature was increased to 220 °C with a rate of 3 °C/min and held for 25 min. The injector temperature was set at 270 ◦C and the detector temperature was set at 300 °C. Individual FA methyl esters were identified by comparison with a standard mixture of 52 Component FAME Mix (Nu-Chek Prep Inc., Elysian, MN, USA).

### 2.8. Statistical Analysis

The chemical evaluations were performed in triplicate and data are reported as mean values. Statistical analysis of compositional data was performed by one-way ANOVA (CoStat, Cohort 6.0), and means separation by the Tukey’s HSD test at *p* ≤ 0.05 of significance.

Statistical analysis of volatile organic compounds characterization was performed by means of the JMP software package (SAS Institute, Charlotte, NC, USA). In particular, hierarchical cluster analysis (HCA) was carried out using Ward’s method [55], with squared Euclidian distances as a measure of similarity on unscaled data. The data matrix was constituted by the complete volatile profiles.

Sensory analysis results were processed by Big Sensory Soft 2.0 (version 2018). In particular, sensory data were analyzed by two-way ANOVA with panelists and samples as main factors [53].

Partial least squares regression (PLS regression) was applied to sensory data in order to define the correlation among quantitative and hedonic parameters, using XLSTAT version 2019.4.1 (Addinsoft Inc. 244 Fifth Avenue, Suite E100, New York, NY, USA, 10001).

## 3. Results

### 3.1. Physico-Chemicals Parameters

As showed in Table 4, no significant effect was observed with regard to water activity and dry matter %, while the free acidity significantly increased accordingly with the rising percentage of the flaxseed cake used for fortification. This evidence suggests that the increasing percentage of flaxseed cake and, consequently, of oil rich in unsaturated fatty acids could significantly promote the quality decay of the fortified breads.

As shown in Table 5, the production of the main fermentative metabolites [56] appears not to be deeply influenced by the flour composition, thus indicating that the biochemical fermentation pathways of the sourdough microflora do not seem significantly affected by the addition of flaxseed cake in the range of the fortifications tested.

### 3.2. Nutraceutical Parameters

The breads baked with different percentages of flaxseed cake (5.0%, 7.5%, 10.0%) and the control were analyzed with the same assays used for the characterization of the flaxseed cake and flour, and the obtained results are reported in Table 5. The nutraceutical value, attributed to baked bread in terms of both total phenols and flavonoids, significantly increased (*p* < 0.001) with the growing percentage of flaxseed cake added to the flour mix. The same trend was observed for the antioxidant power (Table 5).

In relation to fatty acids composition, saturated fatty acids (SFA) were already significantly decreased at 5.0% of fortification with flaxseed cake addition, remaining consistent thereafter. At the same time, an increase in the percentage of both monounsaturated fatty acids (MUFAs) and polyunsaturated fatty acids (PUFAs) n-3 were observed. This is in accordance with the profile of individual fatty acids since the breads obtained with the addition of flaxseed cake also contained higher levels of n-3 alpha-linolenic acid in comparison with the control.

Accordingly, the n-6/n-3 ratio significantly decreased from the control to the different amounts of cake addition, reaching the lowest value at the highest cake fortification amount (Table 6).

### 3.3. Color Determination

As reported in Table 7, the color of the crumb appears significantly influenced by the percentage of flaxseed cake utilized: while Bread 1 showed the higher values of both lightness (L*) and blue–yellow components (b*), the red–green components (a*) significantly increased as a function of the concentration of brown flaxseed flour.

Furthermore, when the metric distances among the chromatic coordinates were calculated (Table 8), not only the color of the crumb of Bread 1 was completely different ($∆E_{ab}^{*}$ >12) from all the fortified crumbs, but it was also possible to discriminate between all the fortified breads among them; in particular, the greatest difference was detected between Bread 4 and Bread 2.

### 3.4. Volatiles Bouquet in the Headspace Emissions of the Cooked Breads

As showed in Table 9, the GC–MS analysis permitted us to identify 51 compounds among the volatile spontaneously released by the eight samples, accounting for 99.1%–99.9% of the total emissions. Among them, small amounts of monoterpenes were present, together with some nitrogen derivatives. However, non-terpene compounds, in particular, aliphatic acids, carbonyl compounds, alcohols and esters, dominated the emission. The main chemicals of the various samples depend on the nature of the sample itself.

In detail, acetic acid characterized the bread prepared using sourdough, particularly when the flaxseed cake was not added to the dough. Indeed, the release of acetic acid percentage decreased with the increasing percentage of flaxseed cake added, but fortified breads are actually characterized by a more complex VOC composition.

Among esters, the emission of ethyl acetate seems, on the contrary, to be directly related to the presence of flaxseed cake in the batter: it was at its minimum in the control sample (Bread 1), while its percentage increased with the amount of the cake. A similar trend was observed for 2-butanone and isobutyl alcohol, both completely absent in Bread 1

**Figure 2:**
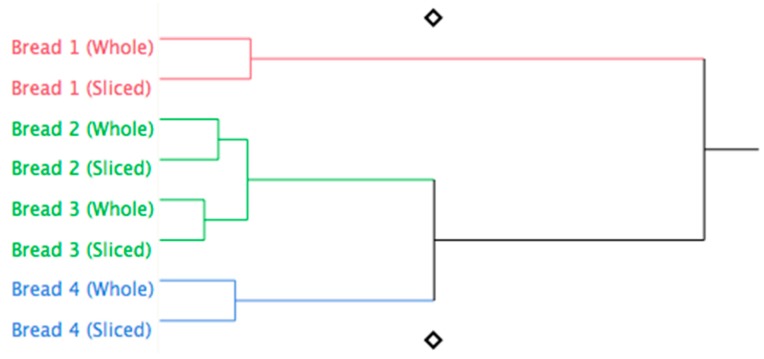
Hierarchical cluster analysis based on volatile compounds of breads (whole or sliced) as a function of % of flaxseed flour used for fortification.

Furthermore, the hierarchical cluster analysis on the volatile aroma compounds (Figure 2) identified four statistical units as a function of the bread’s formulation (Table 2), regardless if whole or sliced bread was analyzed, thus indicating that in the experimental conditions adopted, the main effect was played by fortification.

### 3.5. Sensorial Parameters

On the basis of the two-way ANOVA calculated for all the parameters evaluated during tasting sessions (Table 10), the differences highlighted for both quantitative and hedonic parameters were significant for most of them, with the panelists and percentage of flaxseed cake used for fortification as main effects.

**Figure 3:**
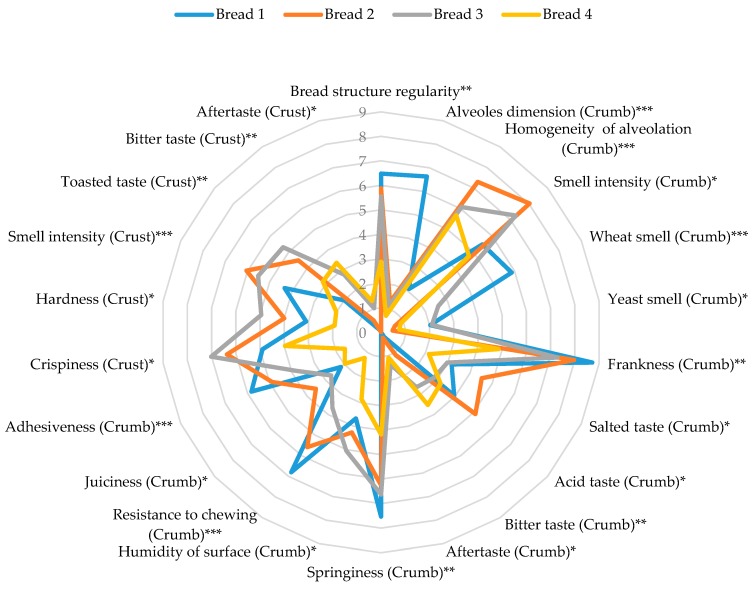
Sensory profile of cooked breads. Significance level *** *p* < 0.001, ** *p* < 0.01, * *p* < 0.05.

In particular, in Figure 3, the mean values assigned to the quantitative parameters that showed a level of reliability are reported.

The sensory profile of the cooked breads in terms of quantitative parameters appeared deeply influenced by the degree of fortification with flaxseed cake, with the main effect shown for the rheological properties of crumb (i.e., dimension and homogeneity of alveolation; adhesiveness and resistance to chewing) as well as the smell intensity and complexity of both crumb and crust (Figure 3).

When the percentage of flaxseed cake used for fortification did not exceed 7.5% (see Breads 2 and 3), the organoleptic expression of fortified breads appeared improved by fortification if they were compared with traditional sourdough bread (see Bread 1). On the contrary, the worst sensory profile was attributed to Bread 4: when 10% of flaxseed cake was used for fortification, all the quantitative parameters evaluated by panelists appeared significantly worsened, with particular attention given to the frankness of the crumb’s smell.

**Figure 4:**
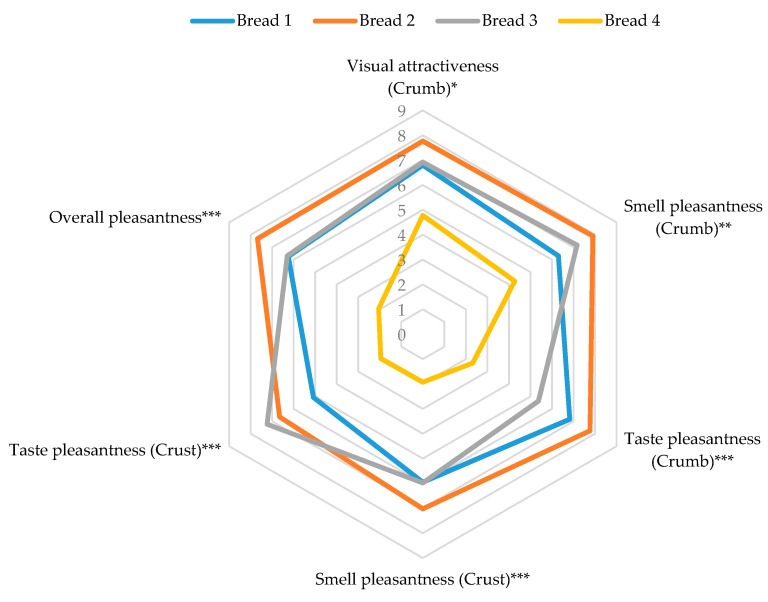
Hedonic profile of cooked breads. Significance level *** *p* < 0.001, ** *p* < 0.01, * *p* < 0.05.

While the hedonic features of a product are generally evaluated during consumer testing [53], in order to describe the hedonic behavior of the obtained breads, as reported in previous papers [50,57], the panelists were also asked to evaluate some hedonic parameters related to view, smell, and taste of both crumb and crust as well as to visual attractiveness of the whole bread and overall pleasantness (Table 10). The median values of the hedonic scores that showed statistically significant differences are reported in Figure 4.

Bread 1, Bread 2, and Bread 3 showed very similar hedonic profiles, with higher values attributed to Bread 2. On the contrary, the use of a higher percentage of flaxseed cake determined a significant decrease of the ratings attributed by panelists to Bread 4 in terms of the pleasantness of both taste and smell of the crust as well as of the taste pleasantness of the crumb and, consequently, of the overall pleasantness.

As for many other food processes, the challenge in fortified cereal food lies in the ability to combine nutritional and health benefits with good sensory quality [2].

The hedonic quality level of a product is fundamental in determining its acceptability, so it is fundamental to investigate and define which features need to be enhanced or reduced for the improvement of the product itself.

In order to plan a targeted product innovation, with consequent maximization of the effectiveness of the resources involved, the PLS regression among quantitative and hedonic parameters was calculated and, consequently, the role played by each quantitative parameter for the definition of the final degree of pleasantness attributed to the different breads was determined (Table 11).

The quantitative parameters that are mainly positively affected by the hedonic behavior of breads fortified with flaxseed cake appear related to the crumb in terms of smell, taste, and texture as well as the rheological features of the crust.

## 4. Discussion

Bread is one of the most widely consumed foods in the world, with over 9 billion kg (20 billion pounds) produced annually [58]. Bread demand is driven by consumers’ seeking convenient fresh products that provide a source of nutritional value. Consequently, freshness is a key component in consumer acceptability and choice of bread [52]. In this context, the use of sourdough as a leavening agent improves the nutritional and technological properties of bread, even in terms of its shelf life [5]. The results obtained demonstrate that flaxseed cake-enriched sourdough bread can represent a potential vehicle for bioactive compounds, with the possibility of obtaining high-quality products with improved nutritional profile and desired health attributes.

These observations are in accordance with previous studies that investigated the effect of fortification with flaxseed cake on baker’s yeast leavened bread. Kaur et al. [59] found that total phenols, total flavonoids, and antioxidant capacity significantly increased in cookies prepared with flaxseed cake and roasted flaxseed cake. Similarly, Meral and Dogan [60], in a study aimed to evaluate the effect of flaxseed on bread-making quality and the antioxidant properties of breads, found that both total phenols and flavonoids significantly increased with the level of fortification with flaxseed and the highest values were found in bread containing 8% flaxseed. Similar results were observed for DPPH and TEAC.

In relation to fatty acid composition, our results showed that the addition of flaxseed cake determined a change of the fatty acids composition in the final products. De Aguiar et al. [61] obtained the highest PUFA to SFA ratio for a whole wheat bread enriched with a combination of 4% ground flaxseed, 8% whole flaxseed, and 5% flaxseed oil. The considerable increase in alpha-linolenic acid (n-3) share in the fatty acids pool was also found in bread and gluten-free bread baked with the 10%–13% addition of ground flaxseeds [62,63]. According to Table 4, a significant decrease in the n-6:n-3 ratio was also observed together with the raise of the fortification level. These ratios are very encouraging, since a n-6:n-3 PUFA ratio ranging from 3:1 to 1:1 is recommended [64]. Recent studies have shown that dietary imbalance of the n-6:n-3 PUFA ratio, with values between 10 and 25 [65,66], can affect human health, as it can lead to increased production of pro-inflammatory cytokines such as tumor necrosis factor-alpha (TNF-α), interleukin-1 (IL-1), and interleukin-6 (IL-6) and thus excessively augment inflammation [67,68].

On the other hand, the higher content of unsaturated fatty acids in the fortified breads could cause their chemical and sensorial deterioration during storage. In this context, the definition of the best storage conditions to preserve the quality of sourdough bread, differently fortified with flaxseed cake, is currently in progress.

According to the literature [69,70], the baking process generally influences the typical aroma of bread crust, while dough fermentation is fundamental for the development of crumb flavor. Thus, the main differences observed between the whole bread dough samples and the corresponding slices are due to compounds developed because of the Maillard reaction, such as furans, pyrroles, and pyrazines.

As for many other food processes, the challenge in fortified cereal food lies in the ability to combine nutritional and health benefits with good sensory quality [2]. In the experimental conditions here adopted, the formulation utilized to produce Bread 2 appears to be the best choice based on the merging of physical–chemical, nutritional, and sensorial data. Indeed, the obtained results suggest that flaxseed cake could be added to sourdough bread formulation up to levels of 5%, with nutritional advantages and good acceptance, offering, at the same time, a very promising healthy alternative to consumers.

## Figures and Tables

**Table 1:** Physical and chemical characterization of flaxseed cake flour and weak wheat flour: dry matter (dm %), water activity (aw), protein (%), fat (%), total phenols, total flavonoids, anti-radical activity, TEAC, and most representative fatty acids (relative %). Data presented are the mean ± SD (*n* = 3).

	Flaxseed Cake Flour	Weak Wheat Flour Type 0
Dry matter (dm, %)	90.61 ± 0.09	91.55 ± 0.10
Water activity	0.54 ± 0.01	0.26 ± 0.01
Protein (g/100 g)	29.20 ± 0.93	10.00 ± 0.44
Fat (g/100 g)	4.41 ± 0.55	1.00 ± 0.12
Total phenols (mg GAE/g dm)	7.40 ± 0.41	1.56 ± 0.16
Total flavonoids (mg GAE/g dm)	0.90 ± 0.03	n.d.
Anti-radical activity (μmol TE/g dm)	17.49 ± 0.77	n.d.
TEAC (μmol TE/g DW)	8.60 ± 0.02	0.30 ± 0.06
C16:0	7.68 ± 0.24	3.13 ± 0.18
C16:1c9	0.10 ± 0.03	n.d.
C17:0	0.14 ± 0.02	n.d.
C18:0	3.65 ± 0.13	0.45 ± 0.05
C18:1c9	20.44 ± 0.89	6.65 ± 0.56
C18:1c11	0.88 ± 0.05	0.87 ± 0.04
C18:2n6	16.32 ± 1.10	52.64 ± 2.43
C20:0	0.19 ± 0.02	n.d.
C18:3n6	0.22 ± 0.04	n.d.
C18-3n3	49.96 ± 3.63	2.89 ± 0.10
C22:0	0.20 ± 0.03	n.d.
C24:0	0.23 ± 0.03	n.d.
SFA	12.07 ± 0.95	34.71 ± 1.89
MUFA	21.43 ± 0.84	7.70 ± 0.82
PUFA	66.50 ± 4.22	55.53 ± 3.12

**Notes:** n.d. = not detectable; GAE= gallic acid equivalents; TE= Trolox equivalents; TEAC=Trolox equivalent antioxidant capacity; SFA = saturated fatty acids; MUFA = monounsaturated fatty acids; PUFA = polyunsaturated fatty acids.

**Table 2:** Sample codes adopted to define the different formulation tested during the research.

Sample code	% Weak Wheat Flour	% Flaxseed Cake Flour
Bread 1	52.0	0
Bread 2	47.0	5.0
Bread 3	44.5	7.5
Bread 4	42.0	10.0

**Table 3:** Lexicon (terms and definitions) for sensory analyses of sourdough flaxseed cake bread products developed in this study ([51] modified).

Parameter	Definition	Portion	Reference *
Quantitative Parameters			
Bread structure regularity	Uniformity of the surface of the sample (visual assessment)	Whole bread	0—high presence of cracks and cuts on the surface/9—regular surface
Alveoli dimension (Crumb)	Size of the pores in the crumb	Slice/Crumb	0—sandwich bread/9—sourdough bread
Homogeneity of alveolation (Crumb)	Homogeneity of the pores in the crumb	Slice/Crumb	0—sourdough bread/9—sandwich bread
Smell intensity (Crumb)	Quantity of odorants compounds as perceived by the assessor	Slice/Crumb	0—absent/9—maximum **
Wheat smell (Crumb)	The aroma associated with wheat flour	Slice/Crumb	0—absent/9—maximum **
Yeast smell (Crumb)	A fermented yeast-like flavor	Slice/Crumb	0—absent/9—maximum **
Pungent smell (Crumb)	The sour aroma associated with vinegar	Slice/Crumb	0—absent/9—maximum **
Frankness (Crumb)	Absence of any off-flavors in smell of crumb	Slice/Crumb	0—smell completely compromised by the presence of off-flavors/9—no off-flavors
Salted taste (Crumb)	Salty basic taste	Slice/Crumb	0—absent/9—maximum **
Acid taste (Crumb)	Acid basic taste	Slice/Crumb	0—absent/9—maximum **
Bitter taste (Crumb)	Bitter basic taste	Slice/Crumb	0—absent/9—maximum **
Aftertaste (Crumb)	The taste-mouth feel aspects of finish	Slice/Crumb	0—good taste-mouth feel after swallowing in agreement with aroma during chewing/9—bad taste-mouth feel after swallowing
Springiness (Crumb)	Sample recovery after the first bite	Slice/Crumb	0—no sample recovery/9—complete sample recovery
Humidity of surface (Crumb)	With blotted lips, amount of moisture/cooling perceived on surface of sample held between both lips	Slice/Crumb	0—dried surface/9—wet surface
Crumb residual after detachment	Residual quantity of crumb attached to the crust after the separation between crust and crumb	Slice	0—absent/9—maximum *
Resistance to chewing (Crumb)	Toughness of the sample perceived during mastication	Slice/Crumb	0—absent/9—maximum **
Juiciness (Crumb)	Amount of juice produced during chewing	Slice/Crumb	0—absent/9—maximum **
Adhesiveness (Crumb)	Force required to remove completely from palate using tip of tongue.	Slice/Crumb	0—absent/9—maximum **
Crispiness (Crust)	Noise made in the first bite of the sample between the molars (auditory assessment)	Slice/Crust	0—absent/9—maximum **
Hardness (Crust)	Force required to first bite through the sample with the molars	Slice/Crust	0—absent/9—maximum **
Smell intensity (Crust)	Those described for crumb	Slice/Crust	0—absent/9—maximum **
Salted taste (Crust)	Those described for crumb	Slice/Crust	0—absent/9—maximum **
Toasted taste (Crust)	The aromatic associated with toasted notes	Slice/Crust	0—absent/9—maximum **
Bitter taste (Crust)	Those described for crumb	Slice/Crust	0—absent/9—maximum **
Aftertaste (Crust)	Those described for crumb	Slice/Crust	0—absent/9—maximum **
Hedonic Parameters			
Attractiveness of shape	The general impression of the visual features of the whole bread	Whole bread	0—completely negative/9—completely positive
Visual attractiveness (Crumb)	The general impression of the visual features of the crumb	Slice/Crumb	0—completely negative/9—completely positive
Smell pleasantness (Crumb)	The general impression of the smell features of the crumb	Slice/Crumb	0—completely negative/9—completely positive
Taste pleasantness (Crumb)	The general impression of the taste features of the crumb	Slice/Crumb	0—completely negative/9—completely positive
Smell pleasantness (Crust)	The general impression of the smell features of the crust	Slice/Crust	0—completely negative/9—completely positive
Taste pleasantness (Crust)	The general impression of the taste features of the crust	Slice/Crust	0—completely negative/9—completely positive
Overall pleasantness	The whole impression based on all the features evaluated	Whole bread	0—completely negative/9—completely positive

Notes: * References settled by the panel after the consensus panel. ** As defined during training.

**Table 4:** Physical and chemical characterization of cooked breads: water activity (aw), dry matter (dm %), free acidity, most representative fatty acids (relative %). Data presented are the mean of three replicates.

	*p*-Value ^1^	Samples (Cooked Breads)			
		Bread 1 ^2^	Bread 2	Bread 3	Bread 4
Water activity (aw)	ns	0.956 ^a^	0.957 ^a^	0.955 ^a^	0.956 ^a^
% of Dry matter (% dm)	ns	56.9 ^a^	55.7 ^a^	55.4 ^a^	57.9 ^a^
Free acidity (acidity degrees)	***	9.12 ^a^	11.20 ^b^	12.31 ^c^	14.31 ^d^
C16:0	ns	15.22 ^a^	11.05 ^a^	15.26 ^a^	11.84 ^a^
C18:1t9	**	0.48 ^a^	0.57 ^a^	0.82 ^a^	3.28 ^b^
C18:0	ns	1.01 ^a^	0.54 ^a^	1.33 ^a^	1.12 ^a^
C18:1c9	ns	8.80 ^a^	8.88 ^a^	13.37 ^a^	12.44 ^a^
C18:2n-6	**	62.50 ^d^	56.44 ^c^	47.12 ^b^	44.32 ^a^
C18:3n-3	***	3.67 ^a^	19.07 ^b^	17.35 ^b^	23.98 ^b^

Notes: ^1^ Significance level *** *p* < 0.001, ** *p* < 0.01; ns: not significant (*p* > 0.05). In the same row, different letters indicate significant differences among samples. ^2^ For bread formulation, see Table 2.

**Table 5:** Fermentative parameters: concentration of main fermentative metabolites in cooked bread. Data presented are the mean of three replicates.

	*p*-Value ^1^	Samples (Cooked Breads)			
		Bread 1 ^2^	Bread 2	Bread 3	Bread 4
Acetic acid (mmoL/g dm)	ns	0.080 ^a^	0.080 ^a^	0.080 ^a^	0.070 ^a^
D-Lactic acid (mmoL/g dm)	ns	0.014 ^a^	0.015 ^a^	0.013 ^a^	0.011 ^a^
L-Lactic acid (mmoL/g dm)	*	0.040 ^a^	0.055 ^b^	0.054 ^b^	0.050 ^b^
Ethanol (mmoL/g dm)	ns	0.050 ^a^	0.050 ^a^	0.060 ^a^	0.065 ^a^

Notes: ^1^ Significance level * *p* < 0.05; ns: not significant (*p* > 0.05). In the same row, different letters indicate significant differences among samples. ^2^ For bread formulation, see Table 2.

**Table 6:** Effect of flaxseed cake percentage on total phenolic content, total flavonoids, and anti-radical activity in fortified bread baked with sourdough. Data presented are the mean of three replicates.

	*p*-Value ^1^	Samples (Cooked Breads)			
		Bread 1 ^2^	Bread 2	Bread 3	Bread 4
Total phenols (mg GAE/g dm)	***	0.481 ^a^	0.671 ^b^	0.932 ^c^	1.041 ^c^
Total flavonoids (mg CAE/g dm)	***	0.083 ^a^	0.165 ^b^	0.216 ^c^	0.241 ^c^
DPPH (μmoL TE/g dm)	***	0.505 ^a^	1.734 ^b^	2.329 ^c^	2.826 ^d^
TEAC (μmoL TE/g dm)	**	0.265 ^a^	0.760 ^b^	1.249 ^bc^	1.522 ^c^
SFA (g/100 g of fatty acids)	***	20.46 ^b^	12.42 ^b^	17.73 ^b^	13.65 ^b^
MUFA (g/100 g of fatty acids)	**	10.57 ^a^	10.40 ^a^	15.26 ^b^	16.41 ^b^
PUFA n-6 (g/100 g of fatty acids)	***	63.32 ^c^	57.63 ^b^	48.27 ^a^	45.22 ^a^
PUFA n-3 (g/100 g of fatty acids)	***	3.79 ^a^	19.18 ^b^	18.12 ^b^	23.98 ^c^
PUFA/SFA	***	1.94 ^c^	1.19 ^b^	1.16 ^b^	0.83 ^a^
PUFA n-6/PUFA n-3	***	16.80 ^c^	3.01 ^b^	2.66 ^b^	1.89 ^a^

Notes: ^1^ Significance level *** *p* < 0.001; ** *p* < 0.01. In the same row, means with different letters are significantly different for *p* < 0.05, following one-way ANOVA test with linseed cake percentage as variability factor. ^2^ For bread formulation, see Table 2.

**Table 7:** Color attributes. L*a*b* values of the cooked bread samples. Data presented are the mean of three replicates.

	*p*-Value ^1^	Samples (Cooked Breads)			
		Bread 1 ^2^	Bread 2	Bread 3	Bread 4
L*	***	62.8 ^d^	49.4 ^c^	44.0 ^b^	40.8 ^a^
a*	***	−0.7 ^a^	2.84 ^b^	3.2 ^c^	3.5 ^d^
b*	***	17.5 ^c^	13.4 ^b^	12.6 ^a^	13.1 ^a^^b^

**Notes: ^1^** Significance level *** *p* < 0.001; ns: not significant. In the same row, different letters indicate significant differences among samples. ^2^ For bread formulation, see Table 2.

**Table 8:** CIE L*a*b* color differences ($∆E_{ab}^{*}$) among cooked bread samples.

$∆E_{ab}^{*}$	Samples (Cooked Breads)			
	Bread 1	Bread 2	Bread 3	Bread 4
Bread 1		14	20	23
Bread 2			5	9
Bread 3				3

**Table 9:** Complete headspace compositions of cooked breads (whole or sliced) as a function of flaxseed percentage. Data presented are the mean of three replicates.

Constituents	L.R.I.	Bread 11 (Whole)	Bread 1 (Sliced)	Bread 2 (Whole)	Bread 2 (Sliced)	Bread 3 (Whole)	Bread 3 (Sliced)	Bread 4 (Whole)	Bread 4 (Sliced)
acetic acid	603	36.3 ± 0.99	45.4 ± 1.10	7.0 ± 0.56	5.5 ± 0.47	5.1 ± 0.38	4.8 ± 0.26	4.8 ± 0.36	4.8 ± 0.25
2-butanone	604			12.4 ± 0.64	14.6 ± 0.61	18.6 ± 0.57	21.3 ± 0.64	14.1 ± 0.56	16.9 ± 0.57
ethyl acetate	611	13.2 ± 0.61	18.0 ± 0.59	19.1 ± 0.62	25.1 ± 0.64	25.3 ± 0.76	24.8 ± 0.68	12.7 ± 0.46	18.5 ± 0.55
isobutyl alcohol	627			17.7 ± 0.55	18.8 ± 0.76	14.9 ± 0.61	16.1 ± 0.60		
isovaleraldehyde	653	4.1 ± 0.38	3.6 ± 0.47	2.8 ± 0.26	2.5 ± 0.25	2.2 ± 0.30	1.8 ± 0.25	5.4 ± 0.31	5.3 ± 0.26
2-methylbutanal	659	3.3 ± 0.30	2.7 ± 0.31	2.0 ± 0.17	1.5 ± 0.23	1.2 ± 0.15	0.9 ± 0.17	5.5 ± 0.40	5.1 ± 0.26
2,3-pentanedione	699							0.5 ± 0.10	0.1 ± 0.00
*n*-heptane	700	0.9 ± 0.10	1.1 ± 0.15	0.8 ± 0.10	1.2 ± 0.17	1.2 ± 0.15	1.5 ± 0.25	1.1 ± 0.15	2.4 ± 0.21
2-ethyl furan	702			2.4 ± 0.26	1.4 ± 0.15	1.9 ± 0.17	1.5 ± 0.21	3.3 ± 0.35	2.5 ± 0.26
3-hydroxy-2-butanone	707							0.7 ± 0.10	0.3 ± 0.00
isopentyl alcohol	736	4.7 ± 0.38	5.6 ± 0.44	5.5 ± 0.40	6.9 ± 0.47	5.5 ± 0.32	7.8 ± 0.47	1.7 ± 0.17	2.6 ± 0.30
2-methylbutanol	737	1.8 ± 0.26	2.1 ± 0.25	1.5 ± 0.25	1.5 ± 0.20	1.3 ± 0.20	1.9 ± 0.21	1.2 ± 0.17	1.5 ± 0.20
1-methyl-1H-pyrrole	744							1.2 ± 0.21	0.7 ± 0.10
pyrrole	754							6.1 ± 0.35	2.4 ± 0.26
hexanal	802	13.8 ± 0.55	7.1 ± 0.49	8.1 ± 0.59	5.3 ± 0.30	8.1 ± 0.46	5.1 ± 0.40	6.8 ± 0.47	5.4 ± 0.38
dihydro-2-methyl-3(2H)-furanone	811							0.5 ± 0.06	0.1 ± 0.00
ethyl lactate	813	2.6 ± 0.21	2.7 ± 0.31	5.4 ± 0.44	5.7 ± 0.40	3.1 ± 0.29	4.0 ± 0.26	1.5 ± 0.25	1.5 ± 0.21
methylpyrazine	830			0.6 ± 0.10	0.4 ± 0.10			1.4 ± 0.20	0.8 ± 0.12
furfural	834	3.5 ± 0.25	2.7 ± 0.17	3.2 ± 0.26	1.7 ± 0.21	1.9 ± 0.25	1.6 ± 0.21	10.2 ± 0.60	6.1 ± 0.47
furfuryl alcohol	858	1.0 ± 0.17	0.4 ± 0.06	0.4 ± 0.00	0.3 ± 0.00			2.7 ± 0.21	2.5 ± 0.20
1-hexanol	869	1.1 ± 0.13	0.8 ± 0.10	0.8 ± 0.10	0.7 ± 0.06	0.7 ± 0.15	1.0 ± 0.21	0.2 ± 0.00	0.5 ± 0.15
5-methylfuran-2(3H)-one	873							0.6 ± 0.10	0.2 ± 0.00
isopentyl acetate	877				0.2 ± 0.00	0.3 ± 0.00	0.4 ± 0.10		
2-methyl-2-octene	884							3.0 ± 0.23	2.1 ± 0.26
2-heptanone	891	0.3 ± 0.00			0.2 ± 0.00			0.4 ± 0.10	0.2 ± 0.0
*n*-nonane	900							0.8 ± 0.17	0.7 ± 0.15
heptanal	903	0.5 ± 0.10		0.4 ± 0.06	0.3 ± 0.06			0.4 ± 0.10	0.4 ± 0.00
2-acetylfuran	913	0.2 ± 0.00							
2,5-dimethylpyrazine	914			0.4 ± 0.10	0.1 ± 0.06			0.8 ± 0.17	0.4 ± 0.15
γ-butyrolactone	915							0.5 ± 0.10	0.5 ± 0.10
2-ethylpyrazine	916			0.3 ± 0.06	0.1 ± 0.00			1.3 ± 0.21	0.9 ± 0.17
2,3-dimethylpyrazine	923							0.5 ± 0.10	0.4 ± 0.06
α-pinene	941			0.6 ± 0.12	0.3 ± 0.10			0.3 ± 0.06	0.3 ± 0.00
benzaldehyde	963	0.5 ± 0.12	0.5 ± 0.06	0.6 ± 0.10	0.3 ± 0.06		0.4 ± 0.06	0.4 ± 0.00	0.6 ± 0.06
5-methylfurfural	964			0.7 ± 0.17				1.1 ± 0.21	1.0 ± 0.17
1-octen-3-ol	982	0.4 ± 0.12							
2-pentyl furan	992	6.5 ± 0.47	3.7 ± 0.26	4.3 ± 0.32	2.7 ± 0.31	4.0 ± 0.20	2.9 ± 0.31	2.8 ± 0.20	3.9 ± 0.26
2-ethyl-6-methylpyrazine	999							1.1 ± 0.15	1.2 ± 0.20
2-ethyl-3-methylpyrazine	1005							0.8 ± 0.17	0.6 ± 0.20
3-ethyl-1-hexanol	1031	0.8 ± 0.20	0.6 ± 0.10		0.5 ± 0.12	0.6 ± 0.10	0.5 ± 0.15	0.1 ± 0.00	
limonene	1032	0.9 ± 0.17	0.7 ± 0.12		0.3 ± 0.10	0.5 ± 0.12	0.8 ± 0.17	1.1 ± 0.26	4.8 ± 0.35
1,8-cineole	1034	1.1 ± 0.29	0.9 ± 0.10						
phenylacetaldehyde	1045	0.4 ± 0.10	0.2 ± 0.00	0.4 ± 0.00	0.3 ± 0.06	0.4 ± 0.06		0.7 ± 0.10	0.7 ± 0.17
γ-caprolactone	1056								0.2 ± 0.06
*(E)*-2-octenal	1061	0.2 ± 0.00							
linalool	1101						0.2 ± 0.00		0.1 ± 0.00
nonanal	1103	0.8 ± 0.15	0.3 ± 0.00	0.9 ± 0.17	0.3 ± 0.06	2.1 ± 0.26	0.3 ± 0.06	0.4 ± 0.06	0.4 ± 0.06
phenylethyl alcohol	1111	0.2 ± 0.06	0.2 ± 0.06	0.5 ± 0.10	0.4 ± 0.10	0.4 ± 0.00	0.3 ± 0.06		
octanoic acid	1179					0.3 ± 0.06			
furfurylpyrrole	1185			0.4 ± 0.06	0.1 ± 0.00	0.2 ± 0.00		0.5 ± 0.10	0.4 ± 0.06
ethyl octanoate	1197				0.1 ± 0.06				
*Monoterpene hydrocarbons*		*0.9*	*0.7*	*0.6*	*0.6*	*0.5*	*0.8*	*1.3*	*5.1*
*Oxygenated monoterpenes*		*1.1*	*0.9*	*0.0*	*0.0*	*0.0*	*0.2*	*0.0*	*0.1*
*Nitrogen/sulfur derivatives*		*0.0*	*0.0*	*1.7*	*0.7*	*0.2*	*0.0*	*13.4*	*7.6*
*Non-terpene derivatives (total)*		*97.1*	*97.7*	*96.9*	*98.0*	*99.1*	*98.9*	*83.3*	*86.4*
*non-terpene hydrocarbons*		*0.9*	*1.1*	*0.8*	*1.2*	*1.2*	*1.5*	*4.9*	*5.1*
*acids*		*36.3*	*45.4*	*7.0*	*5.5*	*5.4*	*4.8*	*4.8*	*4.8*
*non-terpene aldehydes/ketones*		*27.4*	*17.1*	*31.5*	*27.0*	*34.5*	*31.4*	*47.4*	*42.4*
*non-terpene alcohols/ethers*		*16.7*	*13.4*	*33.1*	*33.2*	*29.3*	*32.0*	*11.7*	*13.7*
*non-terpene esters*		*15.8*	*20.7*	*24.5*	*31.1*	*28.7*	*33.5*	*14.5*	*20.4*
*Total identified*		*99.1*	*99.3*	*99.2*	*99.3*	*99.8*	*99.9*	*98.0*	*99.2*

Colors indicate chemical nature of detected compounds. Italics indicate the total percentage of each class of compound.

**Table 10:** ANOVA calculated for all the parameters evaluated by panelists during tasting sessions.

Parameter Evaluated by Panelists	*p*-Value ^1^	*f*	LSD (Least Significant Difference)
Bread structure regularity	**	4.93	1.98
Alveoli dimension (Crumb)	***	197.18	0.65
Homogeneity of alveolation (Crumb)	***	8.35	2.46
Smell intensity (Crumb)	*	4.12	2.01
Wheat smell (Crumb)	***	15.23	1.95
Yeast smell (Crumb)	*	2.40	1.52
Pungent smell (Crumb)	ns	0.71	2.51
Frankness (Crumb)	**	6.26	1.89
Salted taste (Crumb)	*	3.26	1.39
Acid taste (Crumb)	*	2.92	1.49
Bitter taste (Crumb)	**	6.27	1.75
Aftertaste (Crumb)	*	3.59	1.00
Springiness (Crumb)	**	4.76	1.86
Humidity of surface (Crumb)	*	3.31	1.32
Crumb residual after detachment	ns	2.80	1.02
Resistance to chewing (Crumb)	***	22.08	1.49
Juiciness (Crumb)	*	3.16	1.05
Adhesiveness (Crumb)	***	9.10	1.72
Crispiness (Crust)	*	3.23	2.03
Hardness (Crust)	*	4.14	1.64
Smell intensity (Crust)	***	10.75	1.40
Salted taste (Crust)	ns	2.47	1.75
Toasted taste (Crust)	**	4.93	1.98
Bitter taste (Crust)	**	5.25	2.02
Aftertaste (Crust)	*	2.88	1.14
Attractiveness of shape (Whole bread)	ns	2.28	2.69
Visual attractiveness (Crumb)	*	3.37	1.77
Smell pleasantness (Crumb)	**	6.12	1.62
Taste pleasantness (Crumb)	***	22.24	1.34
Smell pleasantness (Crust)	***	15.32	1.49
Taste pleasantness (Crust)	***	18.44	1.41
Overall pleasantness	***	34.06	1.08

Notes: ^1^ Significance level *** *p* < 0.001 (*f* = 7.10), ** *p* < 0.01 (*f* = 4.43), * *p* < 0.05 (*f* = 2.87); ns: not significant.

**Table 11:** PLS regression calculated for all the quantitative parameters evaluated by panelists during tasting sessions and overall pleasantness. Strong correlations (values > 0.5) are highlighted in grey.

Quantitative Parameters	Overall Pleasantness
Bread structure regularity	0.66
Alveoles dimension (Crumb)	0.24
Homogeneity of alveolation (Crumb)	0.35
Smell intensity (Crumb)	0.73
Wheat smell (Crumb)	0.16
Yeast smell (Crumb)	0.14
Pungent smell (Crumb)	−0.01
Frankness (Crumb)	0.75
Salted taste (Crumb)	0.59
Acid taste (Crumb)	0.46
Bitter taste (Crumb)	0.03
Aftertaste (Crumb)	−0.15
Springiness (Crumb)	0.68
Humidity of surface (Crumb)	0.60
Crumb residual after detachment	0.47
Resistance to chewing (Crumb)	0.61
Juiciness (Crumb)	0.70
Adhesiveness (Crumb)	0.53
Crispiness (Crust)	0.65
Hardness (Crust)	0.51
Smell intensity (Crust)	0.81
Salted taste (Crust)	0.37
Toasted taste (Crust)	0.50
Bitter taste (Crust)	−0.09
Aftertaste (Crust)	−0.24
